# A study of the impact of the COVID‐19 pandemic on equine veterinary care in the UK

**DOI:** 10.1002/vro2.74

**Published:** 2023-11-02

**Authors:** Sarah E. Allen, Daniel G. O'Neill, Jacqueline M. Cardwell, Kristien L. P. Verheyen, David C. Brodbelt

**Affiliations:** ^1^ Department of Pathobiology and Population Sciences Royal Veterinary College Hatfield Hertfordshire UK

## Abstract

**Background:**

During the COVID‐19 pandemic, equine health care in the UK may have been adversely affected due to mandated changes in the delivery of veterinary healthcare and the potential for reduced health‐seeking behaviour.

**Methods:**

Electronic patient records (EPRs) were analysed to describe veterinary activity for all equids under the active care of 20 veterinary practices in the UK in the 12 months before and after the introduction of the first UK lockdown. Pre‐pandemic and pandemic levels of clinical activity were compared. Further comparisons of care, including immediate management and treatment, were made following a detailed review of EPRs from randomly selected subsets of equids under care in four time periods.

**Results:**

All measures of activity and face‐to‐face interaction were lower in the early pandemic period than in the equivalent pre‐pandemic period. Compared to pre‐pandemic, the early pandemic was associated with a decrease in prophylactic care and non‐urgent diagnostic imaging and an increase in systemic non‐steroid anti‐inflammatory prescription. Convenience sampling of veterinary practices may have limited the generalisability of the findings. The quality of EPRs was variable.

**Conclusions:**

While equine veterinary activity was significantly disrupted in the early pandemic period, there was a rapid return to pre‐pandemic levels of activity. Subsequent lockdowns appeared to have had little effect on veterinary care.

## INTRODUCTION

The COVID‐19 pandemic posed unprecedented challenges for the client‐facing veterinary profession. The emergence and rapid global spread of the novel SARS‐CoV‐2 virus saw the UK government introduce a series of stringent national restrictions aimed at reducing virus transmission and preventing infections in the immunologically naïve population. These restrictions included quarantining humans experiencing COVID‐19 symptoms or who tested positive, as well as putting limits on social interaction, non‐essential work activities and travel. Collectively, these restrictions were referred to as ‘lockdown’, with the first UK lockdown introduced on 23 March 2020.^1^

Major changes in the delivery of veterinary healthcare were needed for clinics to work in line with rapidly changing government advice. In the early pandemic, veterinary work was initially limited to urgent care and emergency services that maintained the food supply chain.^2^ Changes to normal working practices included non‐contact consultations, the Royal College of Veterinary Surgeons (RCVS) temporarily permitting the remote prescribing of prescription‐only medicines and, where physical examination was necessary, wearing of personal protective equipment. Veterinary practices also had to cope with reduced staffing levels when individuals were required to self‐isolate or needed to care for others. Consequently, delays in veterinary diagnosis and treatment and the potential for reduced health‐seeking behaviours by horse owners during the pandemic may have adversely affected equine health and welfare.

The Royal Veterinary College‐based VetCompass animal health surveillance programme collates anonymised electronic patient records (EPRs) from horse, small animal and farm veterinary practices in the UK.^3^ Analysis of these records can provide objective insight into changes in veterinary activity and care over time. The aim of this study was to evaluate the impact of the pandemic on equine veterinary activity and care in the UK.

## MATERIALS AND METHODS

The study population included all equids under the active care of 20 UK mixed and equine veterinary practices, participating in the VetCompass programme, between 23 March 2019 and 22 March 2021. Equids were considered under active veterinary care if their EPR included at least one care episode dated within the 2‐year study period. A care episode corresponds to a uniquely dated EPR entry and reflects either a free‐text clinical note, administrative note (such as a quote), measurement (such as bodyweight or height), invoiced item or combination of these.

### Description and comparison of pre‐pandemic and pandemic equine veterinary activity

Details of all care episodes provided to the study population were extracted from the VetCompass database. The total number of equids under active veterinary care and the total number of care episodes per month were calculated. To explore the potential impact of changes to the UK government COVID‐19 guidelines, the pandemic year was divided into six time periods based on key restriction changes during the pandemic (Table 1) and the pre‐pandemic year was divided into the equivalent six time periods in 2019 and 2020.

**TABLE 1:** Details of the six study periods in the pandemic year, including dates and imposed COVID‐19 restrictions.

Study period number	Dates	Comments on COVID‐19 restrictions
1	23 March–10 May 2020	The first UK lockdown was introduced. During this period, British Equine Veterinary Association guidance recommended that while 24‐h emergency services should be maintained, all non‐essential and routine work be stopped.
2	11 May–23 June 2020	People were allowed to return to the workplace if they could not work from home. At this point, professional guidance permitted the undertaking of all equine veterinary work, so long as a risk assessment had been performed and the work was conducted in the risk‐mitigating manner.
3	24 June–4 November 2020	There was further relaxing of restrictions including the 2 m social distancing rule.
4	5 November–2 December 2020	England was placed into a second national lockdown. There was no restriction on equine veterinary activity if it could be performed in a safe manner.
5	3 December 2020–5 January 2021	England left lockdown and returned to a strict three‐tiered system of restrictions.
6	6 January–22 March 2021	England entered a third lockdown. Implemented restrictions were relaxed from 8 March 2021, however, limits on indoor mixing were not lifted until 17 May 2021.

Veterinary activity, within each period, was expressed as a percentage of yearly activity, for example, the number of care episodes (23 March–22 April 2019) per total number of care episodes (23 March 2019–22 March 2020).

Clinical activity was defined as either being face‐to‐face or non‐face‐to‐face. Face‐to‐face activity was assumed if the care episode included a measurement or an invoiced item relating to veterinary attendance, for example, visit or hospitalisation, a procedure or a pharmaceutical licensed for intravenous use. Examples of non‐face‐to‐face activity included administrative tasks (insurance form completion, appointment booking), remote visits and other clinical non‐face‐to‐face activity, such as supply of repeat prescriptions and routine endoparasiticides and reporting of laboratory test results.

Box and whisker plots were constructed to show the distribution of period activity and proportional face‐to‐face activity per practice. Wilcoxon signed rank tests were used to determine the statistical significance of any difference in period activity or proportional face‐to‐face activity between the equivalent pandemic and pre‐pandemic periods. Statistical significance was set at the 5% level.

### Comparison of clinical care provided to random samples of equids pre‐pandemic and during the pandemic

For each of four 2‐month periods (Table 2), a simple random sample of 1000 equids was selected from all equids under active veterinary care during the period of interest. Samples were selected using an online random number generator (RANDOM.ORG— Integer Generator 2021). Sample size calculations indicated that for a population of 6000 equids under active care in a given period, a sample of approximately 756 was required to estimate an indication or treatment with a 10% expected frequency with 2% precision at a 95% confidence level (OpenEpi—Sample Size for Frequency in a Population 2021).

**TABLE 2:** Dates of the four 2‐month periods used to compare clinical care during and before the pandemic.

Period name	Dates
Early pre‐pandemic	23 March–22 May 2019
Late pre‐pandemic	5 November 2019–4 January 2020
Early pandemic	23 March–22 May 2020
Late pandemic	5 November 2020–4 January 2021

The periods of interest represented two phases during the first year of the pandemic when the tightest UK government restrictions were in place and the equivalent periods in the pre‐pandemic year. Electronic patient records for all equids in each sample population were manually reviewed and data on all care provided during the corresponding 2‐month period were extracted. Information obtained included date, nature of care episode (face‐to‐face or non‐face‐to‐face), indication type (administrative, routine or prophylactic care, new clinical problem or existing clinical problem), clinical indication(s), services and treatment provided. Routine or prophylactic care indications referred to episodes of care in horses that were not perceived to have a clinical problem, for example, vaccination, routine dentistry and pre‐purchase examination.

A subset of non‐face‐to‐face care episodes were categorised as remote visits. Remote visits aimed to capture care episodes performed via tele‐ or video‐conferencing, which may more normally have been conducted face‐to‐face. A remote visit was defined as any care episode with evidence of problem discussion but no evidence of face‐to‐face activity. Supply of repeat prescriptions and reporting of laboratory test results were not considered to be remote visits unless the animal's current health status was explicitly documented on the date of the episode. Horse demographic data were also extracted.

For each period of interest, horse demographic data were described using summary statistics. Care episodes corresponding to administrative tasks were excluded and the total number of clinical care episodes within each period of interest was reported. The proportion of clinical care episodes with evidence of face‐to‐face activity was calculated, for each period, with 95% confidence intervals (CIs). The number of care episodes corresponding to remote visits was also reported and expressed as a proportion of all clinical care episodes, with 95% CIs. Common procedures and treatments were described numerically and expressed as a proportion of all clinical care episodes, with 95% CIs.

The total number of clinical indications (individual reasons for veterinary care) and number of indications per care episode were reported. Clinical indications were categorised as routine or prophylactic care versus health problems and then expressed as a proportion of all clinical indications. The most common diagnoses for face‐to‐face care, non‐face‐to‐face care and remote visits were determined. The proportion of routine or prophylactic care indications with evidence of face‐to‐face interaction was calculated, with 95% CI. Similar statistics were calculated for new and existing problems with the Wilcoxon signed rank test used to compare activity in corresponding pre‐pandemic and pandemic periods. Statistical significance was set at the 5% level.

## RESULTS

### Study population

The 20 collaborating veterinary practices were spread across the UK, with 13 (65%) in England, three (15%) in Northern Ireland, two (10%) in Scotland and two (10%) in Wales. When described by species treated, five (25%) practices were horse only and 15 (75%) were mixed, of which five had a dedicated equine department. When described by RCVS Practice Standards Scheme accreditation, four (20%) were equine veterinary hospitals, five (25%) were general equine practices and five (25%) met core equine standards. One practice provided out‐of‐hours services only. Over the 2‐year study period, a total of 46,095 equids were under active veterinary care, with a median of 1794 (interquartile range [IQR] 512–3744; range 202–8203) equids per practice.

### Description of pre‐pandemic and pandemic equine veterinary activity

**FIGURE 1:**
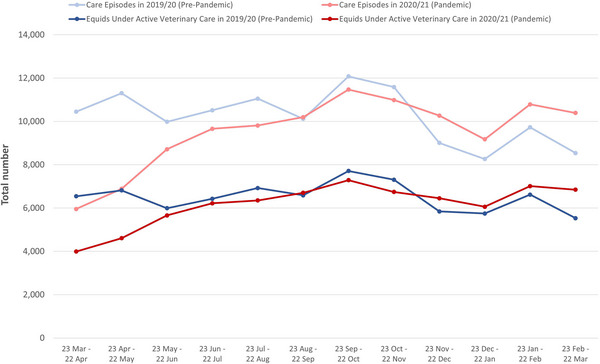
The total monthly numbers of equids under active veterinary care and care episodes for 20 UK mixed and equine veterinary practices between 23 March 2019 and 22 March 2021.

During the study period (23 March 2019–22 March 2021), a total of 236,997 care episodes were provided, with 141,711 (59.8%) having evidence of face‐to‐face activity. The total number of equids under active veterinary care and the total number of care episodes per month are presented in Figure 1. In the month following the introduction of the first UK lockdown (23 March–22 April 2020), there were 2550 (39.0%) fewer equids under active veterinary care and 4492 (43.0%) fewer care episodes compared to the equivalent period in 2019. From approximately 3 months after the introduction of the first UK lockdown, monthly numbers were similar to pre‐pandemic levels.

### Comparison of proportional veterinary and face‐to‐face activity between equivalent pandemic and pre‐pandemic periods

**FIGURE 2:**
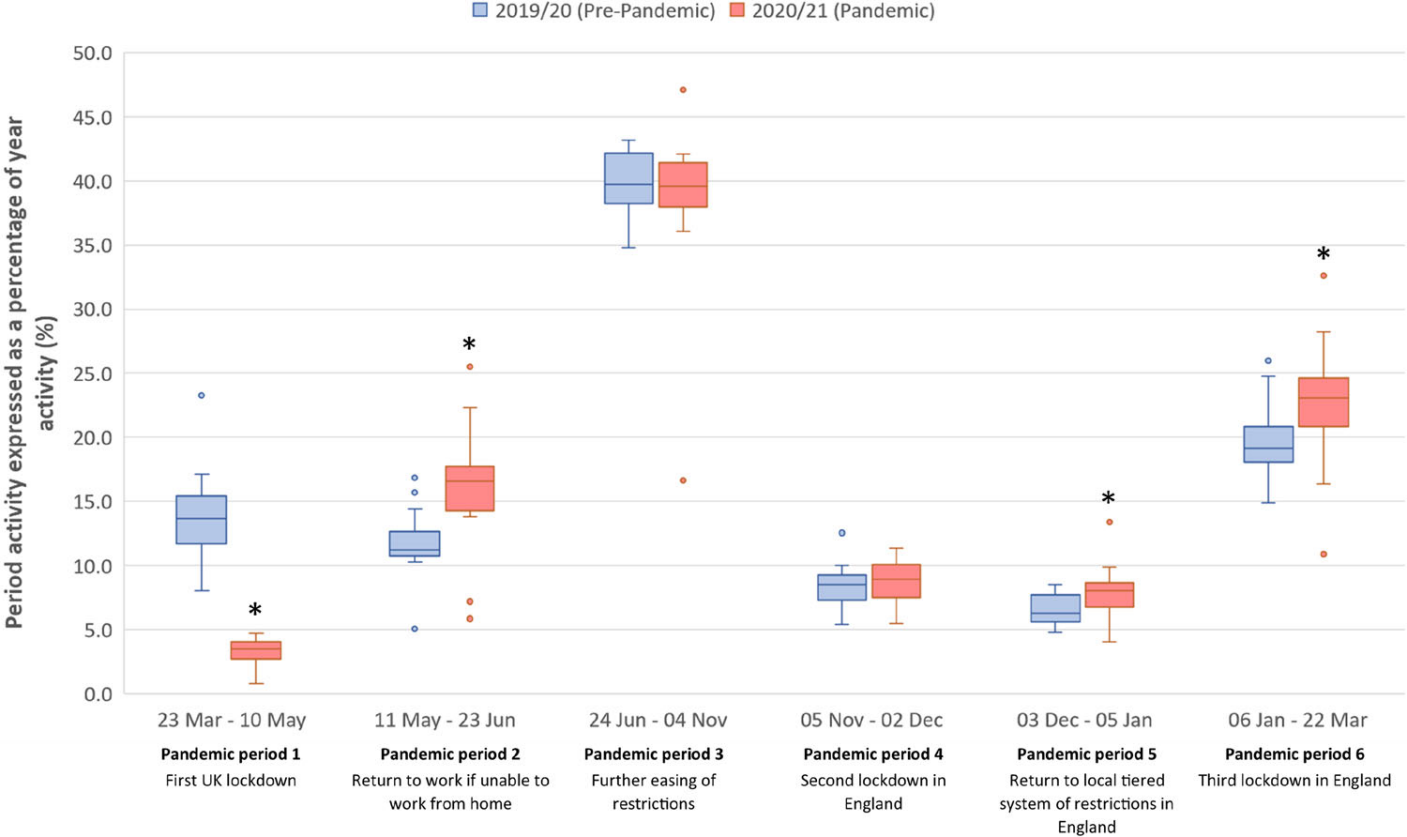
Period activity per practice for 20 UK mixed and equine veterinary practices between 23 March 2019 and 22 March 2021. Asterisk (*) denotes a statistically significant difference between pre‐pandemic and pandemic period activity (*p* < 0.05). Box plots with median values, first and third quartiles and minimum and maximum bars with outliers.

**FIGURE 3:**
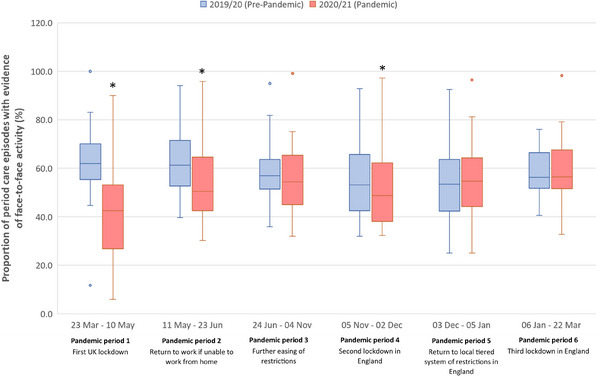
Proportion of period care episodes with evidence of face‐to‐face activity per practice for 20 UK mixed and equine veterinary practices between 23 March 2019 and 22 March 2021. Asterisk (*) denotes a statistically significant difference between pre‐pandemic and pandemic period face‐to‐face activity (*p* < 0.05). Box plots with median values, first and third quartiles and minimum and maximum bars with outliers.

The distributions of period activity (expressed as a percentage of year activity) and proportional face‐to‐face activity per practice are presented in Figures 2 and 3, respectively. During the first pandemic period (23 March–10 May 2020), following the introduction of the first UK lockdown, all practices showed a decrease in activity compared to the corresponding period in the pre‐pandemic year (*p* < 0.001); activity decreased by a median of 10.7% (IQR –12.0% to –8.4%; range –18.9% to –5.9%) per practice. Compared to the equivalent pre‐pandemic period, proportional face‐to‐face activity was also reduced during this time (*p* = 0.001), with a median of 20.2% (IQR –30.6% to –15.0%; range –50.1% to –3.4%) fewer care episodes per practice having evidence of face‐to‐face interaction.

During the second pandemic period (11 May–23 June 2020), immediately after the restriction to work from home was lifted, activity was higher than in the corresponding pre‐pandemic period (*p* < 0.001). Proportional face‐to‐face activity, however, remained lower compared to the equivalent pre‐pandemic period (*p* < 0.001).

During the third pandemic period (24 June–4 November 2020), there was no significant difference in activity (*p* = 0.68) or proportional face‐to‐face activity (*p* = 0.09) compared to the equivalent pre‐pandemic period.

During the fourth pandemic period (5 November–2 December 2020), following the introduction of the second lockdown in England, there was no significant difference in activity compared to the corresponding pre‐pandemic period (*p* = 0.48). However, proportional face‐to‐face activity, during this time, was lower than that observed during the equivalent pre‐pandemic period (*p* = 0.03).

During the fifth (3 December 2020–5 January 2021) and sixth (6 January–22 March 2021) pandemic periods, corresponding, respectively, to when England returned to a tiered system of restrictions based on the risk of COVID‐19 in the local area and the third lockdown in England, activity was greater than that observed in the corresponding pre‐pandemic periods (fifth period, *p* < 0.01; sixth period, *p* < 0.001). For both periods, there was no significant difference in proportional face‐to‐face activity between the pandemic and pre‐pandemic years.

### Comparison of clinical care episodes provided to four random subsets of equids in equivalent pre‐pandemic and pandemic periods

The distribution of demographic data was similar across all four samples. In each sample, the median age was 12 years, and approximately 40%–45% were females and 25%–30% were entire males. The most common breed groups were ponies (19%–25%), thoroughbreds (16%–19%), cobs (15%–18%) and warmbloods (14%–16%), with each group also including the corresponding crosses.

The total number of clinical care episodes provided to the sampled equids was 1781 in the early pre‐pandemic period, 1697 in the late pre‐pandemic period, 1682 in the early pandemic period and 1690 in the late pandemic period. There was a decrease in the proportion of clinical care episodes with evidence of face‐to‐face activity in the early pandemic (*n* = 841, 50.0%, 95% CI 47.6%–52.4%) compared to the early pre‐pandemic (*n* = 1287, 72.2%, 95% CI 70.1%–74.3%) period (*p* < 0.001). There was an increase in the proportion of clinical care episodes defined as remote visits in the early pandemic (*n* = 357, 21.2%, 95% CI 19.3%–23.3%) compared to the early pre‐pandemic (*n* = 212, 11.9%, 95% CI 10.4%–13.5%) period (*p* < 0.001). There was no difference in the proportion of clinical care episodes with evidence of face‐to‐face interaction or defined as remote visits between the late pre‐pandemic and late pandemic periods.

**FIGURE 4:**
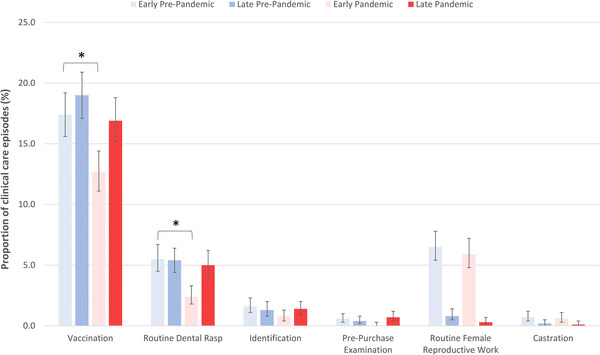
Proportion of clinical care episodes attributable to a selection of routine or prophylactic care activities in random samples of equids under the active care of 20 UK mixed and equine veterinary practices in four periods of interest. The error bars represent 95% confidence intervals. Asterisk (*) denotes a statistically significant difference between the corresponding pandemic and pre‐pandemic periods (*p* < 0.05).

The proportion of clinical care episodes attributed to a selection of routine or prophylactic care activities in each period of interest is presented in Figure 4. The proportion of clinical care episodes attributable to vaccination was lower in the early pandemic (*n* = 214, 12.7%, 95% CI 11.1%–14.4%) than in the early pre‐pandemic (*n* = 310, 17.4%, 95% CI 15.6%–19.2%) period (*p* < 0.001). A decrease in the proportion of clinical care episodes attributed to routine dental treatment was also observed in the early pandemic (*n* = 41, 2.4%, 95% CI 1.8%–3.3%) compared to the early pre‐pandemic (*n* = 98, 5.5%, 95% CI 4.5%–6.7%) period (*p* < 0.001).

There was no difference in the proportion of care episodes attributable to routine female reproductive work, castration, identification or pre‐purchase examination between the early pandemic and the early pre‐pandemic periods. There was no difference in the proportion of care episodes attributable to each of the considered routine or prophylactic care activities between the late pandemic and late pre‐pandemic periods.

**FIGURE 5:**
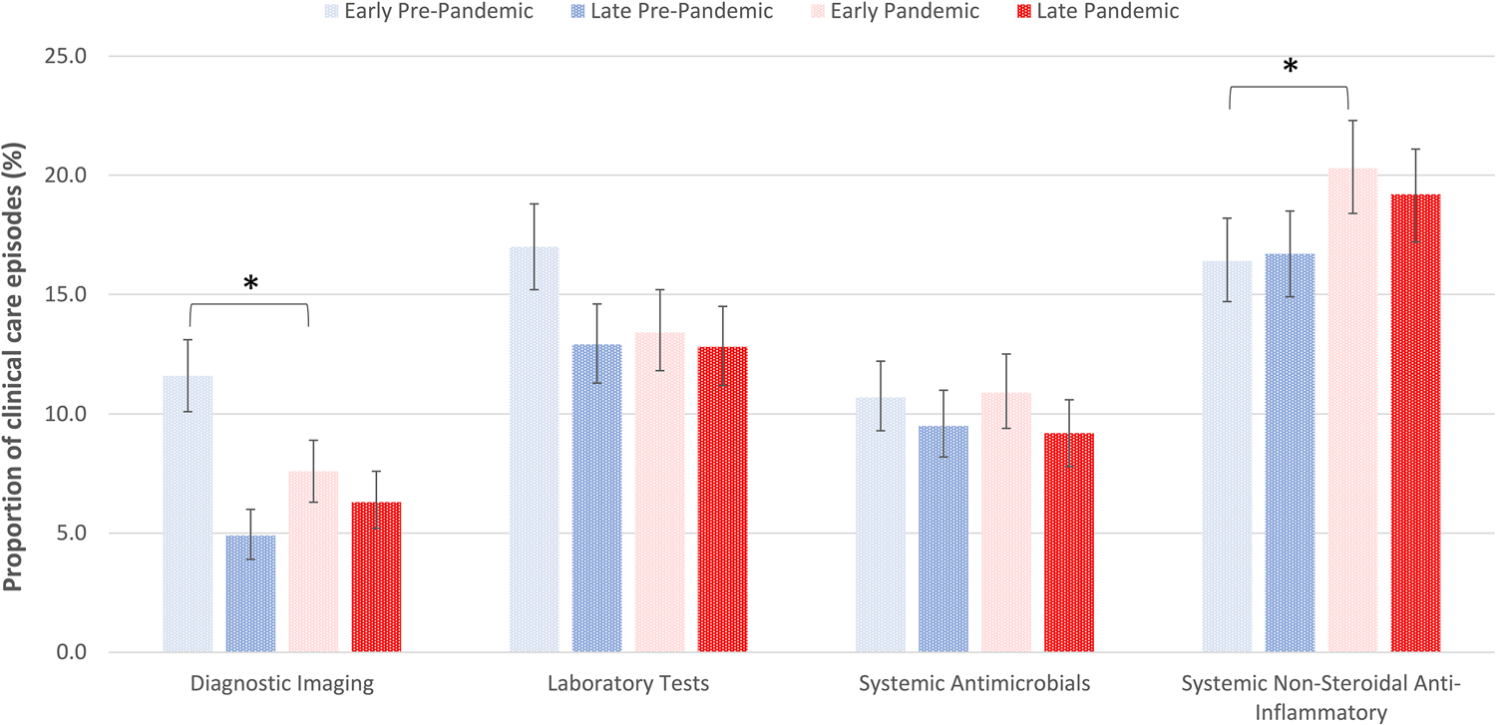
Proportion of clinical care episodes including diagnostic imaging, laboratory testing and prescription of systemic antimicrobials and non‐steroidal anti‐inflammatories in random samples of equids under the active care of 20 UK mixed and equine veterinary practices in four periods of interest. The error bars represent 95% confidence intervals. Asterisk (*) denotes a statistically significant difference between the corresponding pandemic and pre‐pandemic periods (*p* < 0.05).

The proportion of clinical care episodes including selected diagnostic tests and treatments in each period of interest is presented in Figure 5. The proportion of clinical care episodes in which diagnostic imaging was performed was lower in the early pandemic (*n* = 127, 7.6%, 95% CI 6.3%–8.9%) than in the early pre‐pandemic (*n* = 206, 11.6%, 95% CI 10.1%–13.1%) period (*p* < 0.001). There was no difference in the proportion of clinical care episodes associated with diagnostic imaging between the late pandemic and late pre‐pandemic periods. There was no difference in the proportion of clinical care episodes including laboratory testing between either the early pandemic and early pre‐pandemic periods or the late pandemic and late pre‐pandemic periods. The proportion of clinical care episodes with prescription of systemic antimicrobials was similar across all four periods, ranging from 9.2% to 10.7% of clinical care episodes per period. The proportion of clinical care episodes with prescription of systemic non‐steroidal anti‐inflammatory drugs (NSAIDs) was higher in the early pandemic (*n* = 342, 29.3%, 95% CI 18.4%–22.3%) than in the early pre‐pandemic (*n* = 292, 16.5%, 95% CI 14.7%–18.2%) period (*p* < 0.01). The proportion of care episodes associated with euthanasia was similar across all four periods, ranging from 0.8% to 1.6% of clinical care episodes per period.

### Description and comparison of clinical care indications in four random subsets of equids in equivalent pre‐pandemic and pandemic periods

The total number of recorded clinical indications was 1928 in the early pre‐pandemic period, 1880 in the late pre‐pandemic period, 1784 in the early pandemic period and 1842 in the late pandemic period. The number of clinical indications per care episode ranged from 1 to 3 in all four periods, with approximately 85% of care episodes having a single indication.

The proportion of clinical indications associated with routine or prophylactic care was lower in the early pandemic (*n* = 698, 39.1%, 95% CI 36.9%–41.4%) than in the early pre‐pandemic (*n* = 854, 44.3%, 95% CI 42.1%–46.5%) period (*p* < 0.01). In addition, during the early pandemic, the proportion of routine clinical indications with evidence of face‐to‐face interaction was also lower (*n* = 409, 58.6%, 95% CI 54.8%–62.3%) than in the early pre‐pandemic (*n* = 674, 78.9%, 95% CI 76.0%–81.6%) period (*p* < 0.001). No difference in the proportion of clinical indications associated with routine or prophylactic care was observed between the late pandemic and late pre‐pandemic periods.

Across all time periods, the most common indications for face‐to‐face care were wounds (prevalence range: 7.9%–11.9%) and unspecified lameness (prevalence range: 6.4%–13.1%). The most common cases to be managed non‐face‐to‐face were pituitary pars intermedia dysfunction (prevalence range: 15.7%–22.2%) and unspecified lameness (prevalence range: 17.3%–23.3%). Common indications for remote visits included unspecified lameness (prevalence range: 6.5%–13.4%), joint problems (prevalence range: 6.5%–9.2%), wounds (prevalence range: 5.8%–10.4%), laminitis (prevalence range: 4.9%–7.1%) and gastric ulceration (prevalence range: 2.4%–12.9%).

For both new and existing problems, the proportion of clinical indications with evidence of face‐to‐face interaction was lower in the early pandemic (new problem 67.8%, 95% CI 61.8%–73.5%; existing problem 39.4%, 95% CI 35.4%–43.3%) than in the early pre‐pandemic (new problem 93.3%, 95% CI 89.6%–96.0%; existing problem 63.9%, 95% CI 59.7%–68.0%) period. There was no difference in the nature of care provided between the late pandemic and late pre‐pandemic periods for either new or existing problems.

## DISCUSSION

Veterinary EPR data can be used to monitor trends in veterinary activity and prescribing habits. This study describes veterinary activity for 46,095 equids under the active care of 20 mixed and equine veterinary practices in the 12 months before and following the introduction of the first UK lockdown. The early pandemic period was associated with widespread veterinary disruption; however, equine veterinary activity quickly returned to ‘normal’ pre‐pandemic levels and subsequent periods of tightened restrictions appeared to have had little impact on activity.

In the early pandemic period, the UK government enforced stringent measures (collectively known as lockdown) in efforts to mitigate the spread of the COVID‐19 virus.^1^ Professional guidance recommended that all non‐essential and routine veterinary work be delayed and, where physical examination of animals was deemed necessary, that an owner declaration stating they were free of COVID‐19 symptoms be obtained beforehand and social distancing maintained throughout.^4^ This led to a substantial drop in both the number of equids receiving active veterinary care and in the numbers of care episodes provided, although reduced health‐seeking behaviour by owners of equids may also have contributed. A reduction in proportional face‐to‐face activity and increase in remote visits during this time suggests that advice from veterinary professional bodies to conduct consultations via tele‐ or video‐conferencing, where feasible, was implemented. Changes in the nature of care were similar for routine indications, new problems and existing problems.

After restrictions eased in May 2020, absolute numbers of care episodes rose and, initially, there was a significant increase in proportional veterinary activity, likely reflecting efforts to catch up on missed or delayed work. Although many equine veterinary services can fortunately be provided outdoors and in a COVID‐mitigated manner, caution was still being demonstrated, as proportional face‐to‐face activity remained lower than in the equivalent pre‐pandemic period. By June 2020, all absolute and proportional measures of activity had returned to near normal. This is consistent with the findings of the third RCVS COVID‐19 economic impact survey, which reported a marked increase in veterinary practices running a ‘near normal caseload’ at this time.^5^

During the late pandemic period, there was little difference in absolute care episode numbers or proportional activity compared to the late pre‐pandemic period. However, consistent with the requirement to limit social interaction during the second lockdown in England (reduced social interaction was also advised in Northern Ireland, Scotland and Wales), there was a small decrease in proportional face‐to‐face activity. Again, this broadly mirrors the RCVS economic impact survey results, which, for November 2020, reported a 10% decrease in the proportion of normal ‘in‐person services’.^6^

Consistent with guidance that routine 6‐monthly influenza boosters be halted and all other influenza booster vaccinations delayed by 1 month,^4^ the absolute number and proportion of clinical care episodes attributable to vaccination was lower in the early pandemic period than in the early pre‐pandemic period. There was little difference in the absolute number or proportion of clinical care episodes attributable to routine female reproductive work. Initial guidance stipulated that it was difficult to justify offering this service.^4^ However, this guidance was quickly changed, with routine female reproductive work permitted from 10 April 2020, if social distancing could be maintained. Other guidelines stated that breeding is an economically important industry and that stopping veterinary involvement may compromise welfare and seriously disrupt the industry.^4^

In the early pandemic period, there was a decrease in the absolute number and proportion of clinical care episodes attributable to diagnostic imaging compared to the early pre‐pandemic period. Several factors may have contributed to this. Horse owners were discouraged from riding during the first UK lockdown to minimise the pressure on the National Health Service from rider injuries.^7^ This may have concurrently led to fewer equine musculoskeletal injuries, thereby reducing the need for diagnostic imaging. Within the clinical notes, some veterinary practitioners recommended that imaging be delayed until multiple veterinary staff could attend the yard or for the horse to be admitted to the practice, so it could be performed safely and with minimal social interaction.

An increase in the proportion of clinical care episodes where systemic NSAIDs were prescribed was noted in the early pandemic than in the early pre‐pandemic period. The reason for the increased use of NSAIDs in the early pandemic is unclear; however, it may reflect the greater proportion of problem indications managed remotely during this time and potential increased use of trial NSAID courses for minor ailments where face‐to‐face examination was not initially justifiable.

Evidence from this study suggests that, throughout the pandemic, equine veterinary professionals acted appropriately, not only to protect human health but also to ensure that animal health or welfare was not compromised. In addition to the COVID‐19‐mitigated measures described above, there was evidence in the EPRs of veterinary professionals conducting COVID‐19 risk assessments prior to attendance, recommending non‐urgent work such as booster vaccination and routine health checks be delayed and offering non‐certified vaccination, that is, when the vaccine is administered by the owner and the vaccination certificate is not signed by the veterinary clinician. In addition, the clinical narratives often stated that social distancing was maintained and personal protective equipment was worn during physical examinations.

The limitations of this study predominantly relate to the use of secondary EPR data for research purposes. Clinical indication was not recorded for approximately 21% of problem care episodes and the diagnosis was often vague. The collaborating veterinary practices represent a convenience sample that may limit the generalisability of findings, especially given that the majority of practices were in southern England. Although horse demographic data were poorly recorded (only 38.4% of the study population had complete age, sex and breed details recorded), age and breed distributions were comparable to previous reports.^8^, ^9^, ^10^, ^11^ Sex distribution was also compatible with previous work when considered as a binary variable (male/female). However, the proportion of entire males in our study population was higher than expected and likely reflected failure to update an animal's record following castration. The lockdown phases corresponded to those of England and therefore will not have accurately reflected the restrictions imposed upon practices in other parts of the UK—Northern Ireland, Scotland and Wales. That said, as COVID‐19 restrictions were the same across all four nations during the first lockdown and the devolved nations still had some restrictions in place during the second and third lockdowns in England, the impact of differences between them is likely to be small.

In summary, the equine veterinary profession appears to have shown great resilience and adaptability in maintaining essential veterinary services during the COVID‐19 pandemic. While equine veterinary care was disrupted in the early pandemic period, there was a quick return to normal activity.

## AUTHOR CONTRIBUTIONS

All authors were involved in the conceptualisation and design of the study. Sarah E. Allen was responsible for data extraction and performed the analysis. All authors contributed to data interpretation and manuscript preparation and approved publication of this final version. All authors agree to be accountable for all aspects of this work's accuracy and integrity.

## CONFLICTS OF INTEREST STATEMENT

The authors declare they have no conflicts of interest.

## ETHICS STATEMENT

The authors confirm that the ethical policies of the journal, as noted on the journal's author guidelines page. Ethical approval was granted by the Royal Veterinary College (SR2018‐1652). Explicit owner informed consent not required for the retrospective analysis of clinical records. Veterinary practices and groups contributing to the VetCompass programme consented to the sharing and analysis of their de‐identified clinical records for research purposes.

## Data Availability

The datasets generated for this work will be publicly available online at the Royal Veterinary College's Research repository.
